# Truncated RAF kinases drive resistance to MET inhibition in MET-addicted cancer cells

**DOI:** 10.18632/oncotarget.2771

**Published:** 2014-11-15

**Authors:** Consalvo Petti, Gabriele Picco, Maria Luisa Martelli, Elena Trisolini, Enrico Bucci, Timothy Perera, Claudio Isella, Enzo Medico

**Affiliations:** ^1^ Candiolo Cancer Institute - FPO IRCCS, Italy; ^2^ Department of Oncology, University of Torino, Italy; ^3^ Istituto Nazionale Biostrutture e Biosistemi, Roma, Italy; ^4^ Biodigitalvalley Srl, Pont Saint Martin, Aosta, Italy; ^5^ Janssen Research and Development, Oncology Discovery, Beerse, Belgium

**Keywords:** drug resistance, RAF1, BRAF, MET, gastric cancer

## Abstract

Constitutively active receptor tyrosine kinases (RTKs) are known oncogenic drivers and provide valuable therapeutic targets in many cancer types. However, clinical efficacy of RTK inhibitors is limited by intrinsic and acquired resistance. To identify genes conferring resistance to inhibition of the MET RTK, we conducted a forward genetics screen in the GTL-16 gastric cancer cell line, carrying MET amplification and exquisitely sensitive to MET inhibition. Cells were transduced with three different retroviral cDNA expression libraries and selected for growth in the presence of the MET inhibitor PHA-665752. Selected cells displayed robust and reproducible enrichment of library-derived cDNAs encoding truncated forms of RAF1 and BRAF proteins, whose silencing reversed the resistant phenotype. Transduction of naïve GTL-16 cells with truncated, but not full length, RAF1 and BRAF conferred *in vitro* and *in vivo* resistance to MET inhibitors, which could be reversed by MEK inhibition. Induction of resistance by truncated RAFs was confirmed in other MET-addicted cell lines, and further extended to EGFR-addicted cells. These data show that truncated RAF1 and BRAF proteins, recently described as products of genomic rearrangements in gastric cancer and other malignancies, have the ability to render neoplastic cells resistant to RTK-targeted therapy.

## INTRODUCTION

Deregulated RTKs have been implicated in the development and progression of numerous human cancers [1]. Aberrant RTK activation can be caused by different mechanisms, such as autocrine/paracrine stimulation, chromosomal translocations, amplification/overexpression and gain-of-function mutations. In addicted cancers, abnormal RTK activity is required for tumor growth and survival [2]. Hence, in such cases, pharmacological targeting of activated RTKs can profoundly affect growth and survival of tumor cells, leading to clinically meaningful responses in patients [3, 4].

Recently, the Hepatocyte Growth Factor (HGF) receptor MET emerged as a promising RTK therapeutic target in a variety of human cancers [5-7]. Drugs targeting the HGF/MET axis, either small-molecule kinase inhibitors or monoclonal antibodies (mAbs), are currently under clinical testing [8-10]. Copy number gains of the MET gene are particularly frequent in human gastric carcinoma, where protein overexpression occurs in about one quarter of the cases [11]. Gastric cancer cells carrying MET amplification and overexpression have been found to be particularly susceptible to its pharmacological inhibition [12], which corroborates the therapeutic potential of MET inhibition in MET-addicted gastric cancers. However, RTK-targeted therapies are not effective in all cases, and even responsive patients invariably develop secondary resistance, either for compensations in the signalling networks or for activation/overexpression of a different RTK or other cell signalling proteins [13-15]. It is increasingly clear that a better understanding of the resistance mechanisms, along with the identification of novel response modifiers, is crucial to increase the effectiveness of targeted therapy.

**Figure 1:**
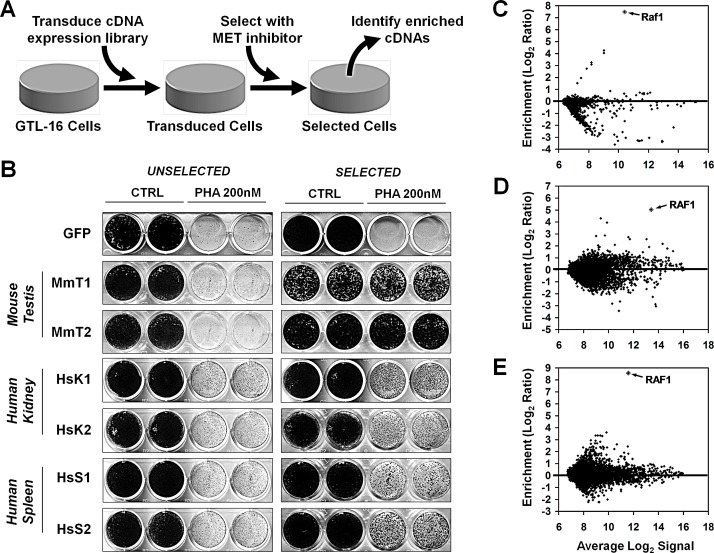
Generation of PHA-resistant GTL-16 cells by transduction with expression libraries (A) Schema of the screening design. GTL-16 cells are transduced with cDNA expression libraries and selected in the presence of PHA-665752 (PHA). Abundance of library-derived transcripts is quantified before and after selection, to identify enriched cDNAs potentially rendering GTL-16 cells resistant to MET inhibition. (B) GLT-16 cells transduced with Mouse Testis (MmT1 and 2), Human Kidney (HsK1 and 2) and Human Spleen (HsS1 and 2) libraries or GFP control vector were treated (SELECTED) or not (UNSELECTED) with 300nM PHA for 8 weeks, after which cells were assayed with 200nM PHA for 2 weeks and subsequently fixed, stained, and photographed. (C,D,E) microarray analysis of library-derived transcripts on GTL-16 cells transduced with (C) mouse testis, (D) human kidney and (E) human spleen libraries. Each MA plot displays average Log2 signal in unselected+selected cells (x-axis), vs. Log2ratio between selected and unselected cells (y-axis) for library-derived mRNAs. Values in MA plots are averaged from two independent transduction-selection experiments.

We therefore set to identify potential mediators of resistance to MET inhibition in cancer cells. There are two main screening strategies to generate drug-resistant cells starting from a sensitive population: (i) induction of spontaneous resistance by long-term drug treatment [16]; (ii) functional screens by transduction of the sensitive cells with cDNA or shRNA libraries [17]. Determinants of resistance to MET inhibition have been successfully identified by long-term drug treatments [18, 19], which however rely on genetic events that could either be pre-existing in a small fraction of cells or occur during the selection process by *de novo* mutagenesis. In both cases the spectrum of identifiable events is limited. We thus performed a complementary screening based on the gain-of-function approach, by which target cells are transduced with full length cDNA expression libraries and then subjected to a selective treatment invariably inducing cell death or growth arrest. Only cells expressing exogenous cDNAs conferring resistance to the treatment will grow and form resistant populations [17, 20]. The model of choice was the GTL-16 cell line, derived from a poorly differentiated gastric adenocarcinoma, in which the MET gene locus is amplified, leading to overexpression of constitutively active MET protein [18]. GTL-16 cells are addicted to MET and respond to small-molecule MET inhibitors with proliferative block and apoptosis [21]. For the screen, GTL-16 cells were transduced with multiple retroviral cDNA expression libraries and selected with the MET inhibitor PHA-665752 (PHA) [21]. The “Xenorarray” approach was then employed to identify, by gene expression arrays, library-derived cDNAs enriched in the selected, resistant populations [22, 23] (Figure 1A).

## RESULTS

### Transduction of GTL-16 cells with expression libraries and selection of PHA-resistant cells

GTL-16 cells were transduced in duplicate with retroviral cDNA expression libraries obtained from Mouse Testis (MmT), Human Spleen (HsS) and Human Kidney (HsK), or with GFP as a control. Microarray-based quantification of library-derived transcripts (see Supplementary Methods) [22] confirmed that all transduced populations carried a consistent number of detectable library-derived transcripts, in addition to a small fraction of background transcripts, also detected in GFP-transduced cells (Supplementary Figure 1). GFP- or library-transduced GTL-16 cells were selected in presence of the MET inhibitor PHA at 300nM for eight weeks. By this time no spontaneous resistance was previously found to occur in non-transduced cells. Cells recovered after selection were assayed for their ability to grow in the presence or absence of PHA. All populations of library-transduced selected GTL-16 cells displayed a significant resistance to PHA compared to unselected counterparts and to both selected and unselected GFP-transduced cells (Figure 1B). These results suggest a biological effect of the library not explained with insertional mutagenesis, but likely deriving from the expression of exogenous transcripts.

### Identification and validation of library-derived cDNAs encoding for RAF1 variants in cells that survived selection with MET inhibitor PHA

To identify cDNAs promoting resistance to PHA, we quantified the abundance of library-derived transcripts in transduced cells before and after PHA selection. In this way, we avoided the need of isolating clones and performing multiple screening cycles. In the case of the mouse testis library, endogenous and exogenous transcripts are from different species, and sequence divergence between orthologue transcripts can be exploited as a “molecular barcode” for species-specific hybridization on microarrays [22]. In the case of human kidney and spleen libraries, we verified that the retroviral vector-specific primer used for reverse transcription (T7-pFB) allows selective reverse transcription of library-derived transcripts (Supplementary Figure 1). In all infections, numerous array probes displayed a higher signal in selected cells compared to unselected, indicating that cells expressing the respective transcripts were enriched by the selection. Many other transcripts were lost, indicating that cells carrying them had died during the selection. To identify the genes that were reproducibly enriched in selected cells, we calculated, for each transcript, the ratio of the array signal before and after selection. Interestingly, the RAF1 transcript showed a strong enrichment in all infections/selections (Table 1 and Figure 1C, D and E).

**Table 1:** Enrichments of library-derived cDNAs in GTL-16 cells transduced and selected for growth in the presence of MET inhibitor Bold values indicate enrichment > 4-fold in at least one of the two screenings

Illumina PROBE_ID	Gene Symbol	Fold Enrichment Screening 1	Fold Enrichment Screening 2
Mouse Testis Library			
scl28523.18.1_12-S	Raf1	**550.5**	**215.7**
scl056407.1_179-S	Trpc4ap	**435.0**	1.0
scl18457.19.17_30-S	Trpc4ap	**319.7**	1.0
scl52811.6_3-S	A930001C03Rik	**20.0**	1.0
scl0113846.1_329-S	V1ra4	**11.4**	0.8
scl32698.6.1_30-S	Irf3	0.8	**312.3**
scl000245.1_108-S	Irf3	0.9	**245.0**
scl00024.1_6-S	Irf3	1.0	**124.9**
Human Kidney Library			
ILMN_1813489	RAF1	**6.7**	**157.6**
ILMN_3251662	HINT3	**4.4**	**10.2**
ILMN_1669663	BCR	**24.2**	1.4
ILMN_3310196	MIR1302-5	**22.4**	1.5
ILMN_1744426	LOC644629	**21.9**	0.8
ILMN_1666966	INS	**18.1**	1.0
ILMN_1664124	FLJ13224	**14.0**	1.4
ILMN_1726466	HDHD3	**13.1**	0.5
ILMN_2321634	RAD17	0.9	**414.2**
ILMN_1687782	RAD17	0.7	**354.2**
ILMN_1771084	ACSM3	0.6	**91.8**
ILMN_1685952	ACSM3	1.1	**44.7**
ILMN_1760858	RAB8A	0.7	**12.8**
Human Spleen Library			
ILMN_1813489	RAF1	**420.8**	**350.3**
ILMN_1794364	CTSW	**11.4**	**8.8**
ILMN_1735594	CDC42SE2	**11.1**	**9.0**
ILMN_2173919	MYO9A	**10.9**	**9.5**
ILMN_1797031	HSPBAP1	**10.0**	3.8
ILMN_1665212	EDC4	**9.1**	**4.6**
ILMN_1777190	CFD	**6.6**	**23.1**
ILMN_1844692	FOXO3	**7.6**	**13.9**
ILMN_1761058	ACAD11	**6.3**	**9.2**
ILMN_1751396	BAG5	**6.6**	**8.9**
ILMN_1745329	PRR14	**5.8**	**8.8**
ILMN_1784766	MCM3AP	**6.1**	**7.0**

**Figure 2:**
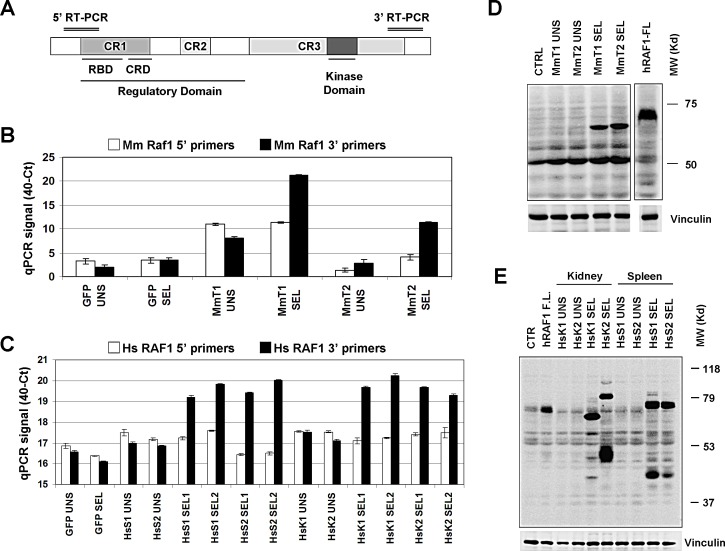
Validation of RAF1 cDNA enrichment (A) Schematic diagram of RAF1 protein domain structure (648 aa) with approximative position of the 5′ and 3′ RT-PCR products. (B, C) Realtime PCR validation of RAF1 transcript enrichment in (B) the GTL-16 Mouse Testis (MmT1 and 2), (C) Human Kidney (HsK1 and 2) and Human Spleen (HsS1 and 2) screenings. The y-axis represents the 40-Ct values, previously scaled against the *PGK1* housekeeper gene. (D, E) Western Blot analysis of RAF1 protein expression in (D) the MmT unselected (UNS) and selected (SEL) cells or in (E) the HsK and HsS unselected (UNS) and selected (SEL) cells, as indicated; GFP transduced cells were used as negative control (CTRL). The less abundant band of ~80kD in the “HsK2 SEL” lane is compatible with an upstream in frame alternative translation start site in the vector sequence. The RAF1 protein was detected by an antibody against the RAF1 C-terminus portion of both human and murine origin. GTL-16 cells transduced with human RAF1 full-length as control for the full size protein detection; vinculin was used as loading control.

We validated RAF1 enrichment in all selections by realtime quantitative PCR (RT-PCR) (see Supplementary Methods). Four pairs of PCR primers were designed, two against murine Raf1 (for the MmT-transduced cells) and two against human RAF1 (for the HsK and HsS-transduced cells), covering the 5′ portion and the 3′ portion of the transcripts (Figure 2A). Surprisingly, only the primer pairs corresponding to the 3′ region confirmed murine RAF1 mRNA enrichment in all selections (Figure 2B and C). Subsequently, Western blot analysis of the enriched populations compared to controls were performed. In the selected, resistant GTL-16 populations, RAF1 antibody detected lower molecular weight bands, compared to the full length control, confirming the hypothesis that the enriched cDNAs encoded for truncated proteins, lacking the N-terminal region (Figure 2D and E).

To verify the composition of the expression libraries before and after PHA selection, we conceived an approach based on deep-sequencing of retroviral library inserts, exploiting targeted reverse transcription (see Supplementary Methods) of RNA obtained from GTL-16 transduced with HsS library, before and after selection, in two independent replicates, using the GS FLX 454 to maximize reads length. The single lanes yielded respectively 420456 and 365867 reads for the two unselected populations and 119949 and 144378 reads for the selected populations. Average read length was around 300 nt. All the reads were mapped with BLAST on RAF1 mRNA sequence to evaluate its abundance and sequence. RAF1 coverage was quite low in unselected cells, and evenly distributed across all the transcript sequence (Supplementary Figure 2A). As expected, RAF1 coverage was much higher in selected cells, with a sharp decline of coverage 5′ from nucleotides 1000-1500 of the RAF1 mRNA in both selected samples (Supplementary Figure 2B). To identify reads compatible with RAF1 truncations, we selected all the reads with matches on both RAF1 and retroviral vector sequence. This analysis revealed that in both selections some reads captured the point of transition from the RAF1 sequence to the vector cloning site, all at the same site of RAF1 (Supplementary Figure 2C). This result shows that, albeit in the original expression library the major form of RAF1 cDNA is full length, PHA selection drives enrichment of a variant that contains the kinase domain, but lacks the N-terminal regulatory domain. No other truncation forms were identified by the analysis, suggesting that this is the most represented form of RAF1 in two independent selections.

To search for truncated forms of RAF1 in published data, we interrogated the ProteinQuest (BioDigitalValley) data mining platform that integrates information from scientific literature, data repositories and biological images. This tool generated an image derived from the sum of all published pictures of SDS-page/Western Blots against the C-terminal portion of RAF1 and highlighted the presence of multiple possible truncated forms of this protein (Supplementary Figure 3 and Supplementary Methods). We then cloned and sequenced RAF1 containing transcripts from all the selected cell populations. In all cases the RAF1 transcripts enriched in selected cells were truncated, at M350 in the MmT, at P308 in the HsK and, as expected, at G248 in the HsS resistant GTL-16 cells (Supplementary Figure 4A).

### Resistance to MET inhibitors of GTL-16 cells expressing truncated Raf1 is reversed by Raf1 silencing

**Figure 3:**
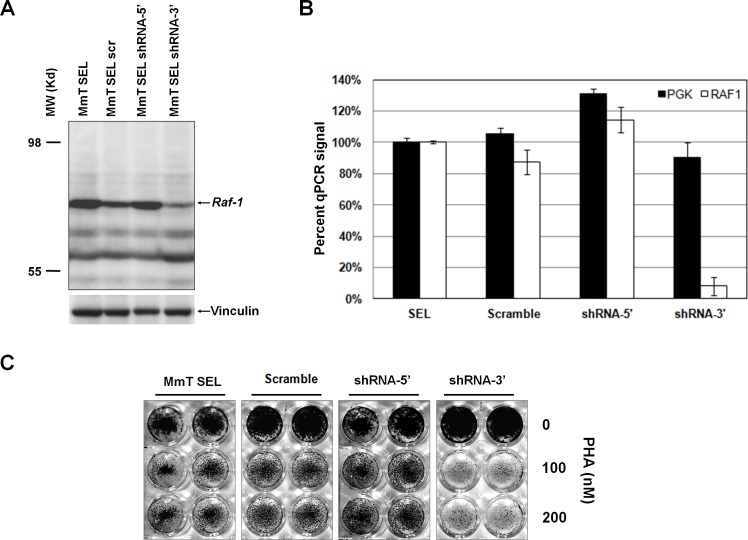
Silencing of truncated murine Raf1 restores sensitivity to PHA (A) Western Blot analysis on resistant Mouse Testis (MmT) GTL-16 cells, transduced with non silencing scramble vector (scr) or two different murine-specific shRNAs against Raf1: shRNA-5′, targeting the 5′ portion of the Raf1 transcript, and shRNA-3′, targeting the 3′ portion. The protein was detected by an antibody against the C-terminus of Raf1 (bands with lower molecular weight respect to the ~60kD truncated RAF1 band are non-specific and appear also in non-transduced cells); vinculin was used as loading control. (B) Realtime PCR validation of murine Raf1 mRNA specific silencing by the shRNA-3′ construct. The y-axis represents the percentage of inhibition in scramble and shRNA-3′ compared to resistant MmT GTL-16 cells (SEL); *Pgk1* housekeeper gene was used as control. (C) Resistant GTL-16 MmT cells, either untransduced (MmT SEL) or transduced with non-silencing shRNA (scramble) or shRNA-5′ or shRNA-3′ silencing constructs were treated with increasing concentrations of PHA (as indicated) for 1 weeks and subsequently fixed, stained, and photographed.

To test whether Raf1 silencing could revert PHA resistance in selected GTL-16 cells, we performed a loss-of-function assay. This could be possible only for MmT-transduced cells, because in these cells the library-derived Raf1 is murine and therefore can be silenced by murine specific shRNAs without interference with endogenous human RAF1. Two different murine Raf1 shRNAs and the non-silencing control lentiviral supernatants were used to transduce MmT-selected, resistant GTL-16 population, and Western Blot analysis showed a good silencing only with shRNA targeting the C-terminal portion of the gene (Figure 3A). RT-PCR analysis confirmed the capability of Raf1 shRNA-3′ to downregulate the expression of Raf1 in MmT-selected GTL-16 (Figure 3B). A growth assay confirmed that resistance of MmT-selected GTL-16 cells to PHA was lost when murine Raf1 was silenced (Figure 3C). These results prove that RAF1 plays a causative role in mediating resistance to MET inhibition.

### BRAF is an additional mediator of PHA-665752 resistance in GTL-16 cells

**Figure 4:**
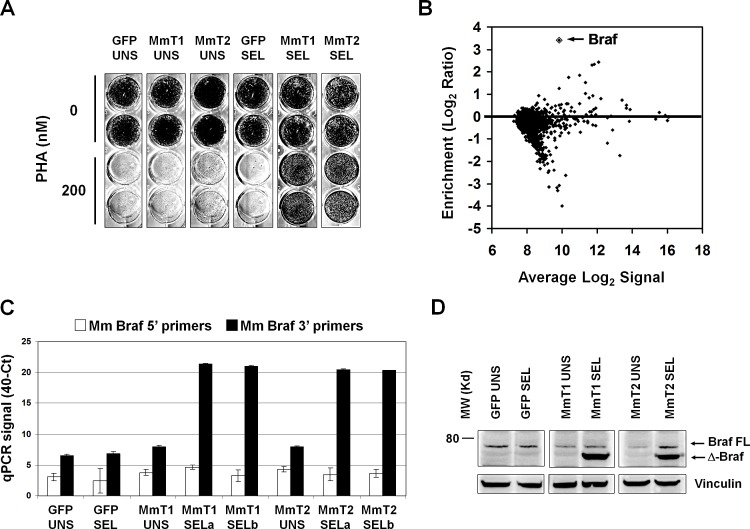
PHA-resistant cells emerging after murine Raf1 silencing are enriched for truncated Braf (A) Growth assay on murine Raf1-shRNA-3′-GTL-16 cells transduced with GFP or mouse testis library. Library-transduced, selected populations (MmT1 SEL and MmT2 SEL) are resistant to 200nM PHA treatment for 2 weeks. (B) MA plot of the xenoarray analysis displaying enrichment of mouse testis library-derived cDNAs after selection (y-axis) vs average Log2 signal (x-axis). Values are averaged from two independent transduction-selection experiments. (C) Realtime PCR validation of enriched murine Braf transcript in GFP and Mouse Testis (MmT1 and 2) selections. The y-axis represents the 40-Ct values, previously scaled against the Pgk1 housekeeper gene. Black and white columns represent, respectively, the 3′ and 5′ RT-PCR products. Only the 3′ RT-PCR confirmed cDNA overexpression in both selections. (D) Western Blot analysis on murine Raf1-shRNA-3′-GTL-16 cells transduced with GFP or mouse testis library (MmT1 and MmT2). Braf was detected by an antibody against the C-terminus portion; vinculin was used as loading control. The full-length (FL), endogenous human crossreacting BRAF, is faintly detectable in all samples.

The data obtained in previous experiments prompted to perform a new screening with this rationale: (i) Darwinian selection of the most resistant cells, carrying truncated Raf1, may have not allowed enrichment of other possible interesting, though weaker, hits; (ii) murine Raf1 shRNA-3′ was found to silence very efficiently the murine Raf1 in infected-selected GTL-16 and to be specific for the murine transcript and therefore not to interfere with endogenous human RAF1 in GTL-16 cells; (iii) therefore, use of GTL-16 transduced with murine Raf1 shRNA-3′ as recipient cells for the MmT library should allow additional weaker candidates to emerge upon PHA selection. Accordingly, wild-type GTL-16 cells were first transduced with murine Raf1 shRNA-3′ and then with the mouse testis library or GFP, in duplicate. After 8 weeks of in PHA selection under the same conditions described above, only library-infected, selected cells became resistant to PHA (Figure 4A), although to a lower extent that the previously selected cells. In both independent selections, xenoarray analysis showed that the murine cDNA enriched in selected cells was Braf (Figure 4B). We performed PCR validation using two oligonucleotide pairs, one corresponding to the 5′ and the other to the 3′ portion of the Braf transcript and, again, only the C-terminal primer pair confirmed cDNA enrichment in both selections (Figure 4C). Western Blot analysis demonstrated that also the Braf protein is truncated in selected cells (Figure 4D). We then cloned and sequenced the enriched Braf transcript, and found a truncated transcript starting at M475 (Supplementary Figure 4B). Therefore, also in this case only the C-terminal portion, containing the kinase catalytic domain of Braf is expressed in resistant cells, while the N-terminal portion, containing regulatory domains, is missing.

### Truncated but not full length RAF kinases confer resistance to MET inhibition, which is reversed by MEK1/2 inhibition

**Figure 5:**
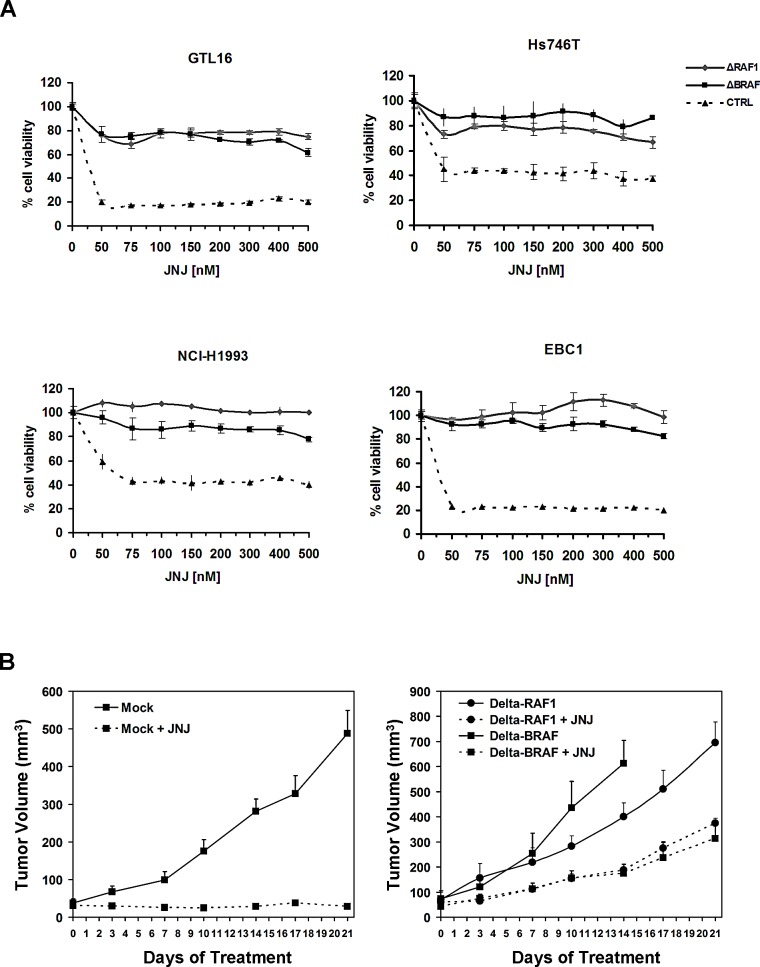
Expression of truncated RAF1 or BRAF confers resistance to MET inhibition and *in vito* and *in vivo* (A) Cell-viability assay at different concentrations of JNJ on GTL-16, Hs746T, NCI-H1993 and EBC1 cells (as indicated) transduced with truncated RAFs or control vector. (B) Tumor growth curves of xenografts from GTL-16 cells transduced with the control vector (left panel) or truncated RAFs (right panel) in nude mice treated with JNJ (40 mg/kg/day) or vehicle.

To confirm whether RAF1 and BRAF were drivers of resistance to MET inhibition, we transduced full length human RAF1 and BRAF in wild-type GTL-16 cells. RT-PCR and Western Blot analysis confirmed robust expression of both RAF1 and BRAF transcripts and proteins (Supplementary Figure 5A and B). However, a growth assay performed on transduced and control cells did not show resistance to PHA nor to JNJ38877605 (JNJ, a novel “second generation” MET inhibitor) [24] in a range of concentrations (Supplementary Figure 5C and D). It appeared therefore likely that both RAF1 and BRAF confer resistance to MET inhibition only when lacking the N-terminal regulatory portion. We therefore transduced GTL-16 cells with truncated human RAF1 and BRAF, resembling those actually observed in human tumors (Supplementary Figure 4). RT-PCR and Western Blot analyses (Supplementary Figure 6A and B) confirmed robust expression of truncated transcripts and proteins. We found that cells expressing truncated RAFs were resistant to the MET inhibitors JNJ (Figure 5A) and PHA (Supplementary Figure 7). We reasoned that RAF blocking drugs would be able to restore sensitivity to PHA in GTL-16 cells expressing truncated RAFs. However, RAF1 selective inhibitors are not available, and truncated forms of BRAF have been previously associated with resistance to the corresponding inhibitors [25]. We therefore decided to target MEK, a downstream effector of RAF proteins. GTL-16 cells expressing truncated RAF1 or BRAF were therefore tested for sensitivity to the MEK1/2 inhibitor AZD6244, either alone or in combination with PHA. While ineffective alone, AZD6244 reverted RAF-driven resistance to MET inhibition in GTL-16 cells (Supplementary Figure 8)

To evaluate whether resistance to MET inhibition in GTL-16 cells transduced with truncated BRAF or RAF1 was also maintained *in vivo*, we performed xenograft experiments in nude mice. Since PHA has poor pharmacokinetic properties and low oral bioavailability [21], we treated the grafted nude mice with the orally available JNJ (40mg/kg/day). JNJ administration exhibited a prominent cytostatic effect on tumors obtained from GTL-16 control cells, whose growth was halted. Conversely, xenografts from cells transduced with either of the two truncated RAFs continued growing despite treatment with the MET inhibitor (Figure 5B). These results show that expression of truncated RAF kinases renders GTL-16 cells resistant to MET inhibition also *in vivo*.

### Truncated RAFs confer resistance to RTK inhibition in different tumor types

To investigate whether truncated RAF1 and BRAF could drive resistance to RTK inhibition also in other cell lines and tissues, Hs746T (gastric carcinoma, MET addicted), NCI-H1993 (non-small cell lung cancer, MET addicted), EBC1 (lung squamous cell carcinoma, MET addicted) and NCI-H508 (caecal adenocarcinoma, EGFR addicted), were transduced with truncated RAFs and GFP control vector. RT-PCR and Western Blot analysis confirmed truncated RAFs expression at high levels (Supplementary Figure 6A and B). As shown in Figure 5A, all MET-addicted cells acquired resistance to MET inhibition by JNJ when transduced with either of the truncated RAFs. Identical results were obtained when PHA was used to inhibit MET, or when the EGFR targeted drug Cetuximab was used on NCI-H508 cells (Supplementary Figure 7). These data show that resistance to RTK inhibition can be induced by truncated RAF1 or BRAF in multiple cell lines, of different origin (stomach, lung and intestine) and addicted to different RTKs (MET, EGFR).

## DISCUSSION

The HGF-MET axis, tightly regulated under normal conditions and involved in epithelial cell proliferation and motility during development, appears to play an important role in oncogenesis, particularly in the development of the invasive and metastatic phenotypes [5]. MET inhibition is an attractive opportunity for molecularly targeted cancer therapy [6, 7]. However, intrinsic or acquired resistance can lead to limited therapeutic effectiveness and disease progression. Similar to other RTKs, development of resistance to MET inhibitors may occur via MET amplification [26] or sequence mutations preventing drug binding [27]. It has also been previously reported that the activation of EGFR family receptors [28], or KRAS, BRAF or AKT [29] can bypass MET inhibition. Indeed, several drugs have been developed to inhibit MET protein, but the mechanisms of resistance have not been fully elucidated [5].

To anticipate potential resistance mechanisms, we carried out an *in vitro* functional screening to identify genes conferring MET inhibitor resistance to GTL-16, a MET-addicted gastric carcinoma cell line with high level amplification of the *MET* gene locus. Here, we report the identification of truncated RAF1 and BRAF as strong determinants of resistance to MET inhibition in GTL-16 cells. The experimental approach described above is extremely reproducible. We identified truncated forms of RAF genes in three different cDNA libraries of two different species, and results were confirmed in *in vitro* and *in vivo* models.

It must be acknowledged that the truncated variants of RAF genes found by our screens are likely to derive from cDNA cloning artifacts. It is possible that the source of truncated transcripts could be attributed to secondary mRNA structures in the phase of reverse transcription prior to cloning cDNAs in the retroviral expression library. However, truncated and fusion RAF1 and BRAF transcripts have been found to occur in several cancer cell lines and tumor samples [30-35]. Recently, long-term treatment of GTL-16 cells with a MET inhibitor was found to generate spontaneously resistant cells. A fusion gene coding for a truncated BRAF protein was identified in the resistant population, although not functionally validated as the driver of the resistance [19]. In our work, we find that RAF1 could play a significant role in resistance to RTK-targeted treatment. To our knowledge, such a causal relationship has not been reported before.

The functional and biochemical properties of RAF truncated variants have been extensively studied since the discovery in 1983 of a viral oncogene encoding a RAF1 kinase lacking the N-terminal domain [36]. Indeed, lack of the N-terminal RAF regulatory domains is known to render RAF kinases constitutively active [37]. In the past, several studies supported the ability of truncated RAF1 to induce transformation in fibroblasts [38-40], but only recently many aberrant forms of RAF genes have been described and associated to cancer progression. The presence of BRAF and RAF1 fusions has been reported to activate mitogen activated kinase-like protein (MAPK) in pilocytic astrocytoma [34]. Another study found rearrangements in gastric, prostate cancer and melanoma that involve again fusions containing BRAF or RAF1 segments [41]. All these rearrangements were characterized by 5′ regulatory elements of highly expressed genes fused to the C-terminal portion of BRAF or RAF1 gene. As an example, in prostate cancer rearrangements occurred between untranslated exon 1 of SLC45A3 (a prostate-specific androgen responsive gene) and exon 8 of BRAF (GenBank GU149303) or between exon 13 of ESRP1 (an epithelial splicing regulatory protein) and exon 6 of RAF1 (GenBank GU149302). All these alterations involve the loss of N-terminal regulatory domain of RAFs, indicating that the aberrant proteins are constitutively active. Intriguingly, in GTL-16 cells selected with the MET inhibitor we have found a murine truncated Braf sequence that is homologous and share exactly the same predicted open reading frame of a rearranged BRAF found in human prostate cancer.

In addition to genomic rearrangements in cancer, RAF proteins may be physiologically truncated by proteolytic cleavage as part of cell survival pathways. In particular, cleavage of RAF1 by caspase-9 was found to protect hematopoietic cells from IL-3 withdrawal-induced apoptosis [42]. Also in this case the resulting truncated RAF1 lacks the N-terminal domain, so that the C-terminal kinase domain can directly interact with the mitochondria to inhibit apoptosis. A similar mechanism could enable cancer cells to escape apoptosis induced by RTK-targeted drugs.

The identification of translocations affecting RAF genes in prostate, gastric cancers and melanoma provided evidence for the key role of RAF signaling in a subset of these cancers and suggested possible new personalized cancer therapy. Several studies identified activated RAF proteins as possible candidates for drug treatment [43], but also highlighted possible mechanisms of resistance to RAF inhibition [37, 44, 45], including truncations [25, 46]. In principle tumors with activated RAFs but resistant to their inhibition should be sensitive to inhibitors of downstream hubs in the same pathway, such as MEK inhibitors. Indeed, in our model, the MEK inhibitor AZD6244 abrogated RAF-driven resistance of GTL-16 cells to MET inhibition. This study therefore indicates a role of RAF truncations in resistance to RTK-targeted therapy, and provides a rationale for future testing of combinatorial treatment approaches.

## MATERIALS AND METHODS

### Cell Culture, Reagents, viral transduction and drug selection

GTL-16 cells, not commercially available, were a kind gift from Silvia Giordano, University of Torino; EBC1 cells were obtained from the JCRB cell bank; Hs746T, NCI-H1993 and NCI-H508 cell lines were obtained from American Type Culture Collection (ATCC). Commercial cells were used within 6 months from their arrival. All cells were regularly authenticated by the Promega Cell ID system, which is based on sequencing known STRs (short-tandem repeats). GTL-16, EBC1 and Hs746T cells were cultured in Dulbecco's modified Eagle's medium (DMEM) (Gibco). NCI-H1993 and NCI-H508 cell lines were cultured in RPMI-1640 medium (Gibco). The medium was supplemented with 10% FBS (Sigma) in a humidified atmosphere of 5% CO2. Human full length RAF1 cDNA was purchased from RZPD German Resource Center for Genome Research and cloned into the pMSCV retroviral vector (Stratagene). Full-length human BRAF cDNA in retroviral pBabe vector (Addgene) was a gift by Federica Di Nicolantonio. Retroviral supernatants were produced in Phoenix Amphotropic packaging cells. The pMSCV-empty retroviral and supernatant was used as control. Human truncated RAF1 and BRAF cDNAs were custom-synthesized by Geneart and cloned into the pRRLsin-PPThCMV-wpre lentiviral vector [47]. All vectors above described were sequenced to confirm truncated RAFs cloning. To downregulate the murine Raf1 transcript, two lentiviral short hairpin RNAs (shRNA) of the Mission shRNA Target Set (Sigma) were used, named shRNA-5′ for N-Terminus portion (TRCN0000055139), shRNA-3′ for C-Terminus portion (TRCN0000055141) and pLKO.1-puro (cat. num. SHC001) non silencing control vector. All lentiviral supernatants were produced by Lipofectamine transfection of HEK293T cells. Transduction procedures, described in detail in Supplementary Methods, were followed by selection with puromycin (2 ng/ml) for retroviral vectors and hygromycin (400ug/ml) for lentiviral vectors. MET small-molecule inhibitors for *in-vitro* assays, PHA-665752 and JNJ38877605, were obtained, respectively, from Sequoia Research Products and Johnson&Johnson; JNJ38877605 for *in-vivo* experiments from Timothy Perera; the EGFR inhibitor Cetuximab from the hospital pharmacy at our Institution; MEK inhibitor AZD6244 from Sequoia Research Products.

### Growth and cell-viability assays

For growth assays, 10^4^ cells of each cell line were seeded in duplicate in 24-well plates. Cells were treated with different drug concentrations. The stain was performed with crystal violet after cell fixing with a solution of 3% paraformaldeide and 1% glucose. Cell viability was measured after 3, 7 and 14 days. Cell viability was determined seeding 1,5 × 10^3^ cells in 100 μl of final volume for each well, in a 96-well plate. Cells were cultured in the specific medium with 10% FBS (Sigma). The next day cells were treated at increasing drug concentrations. After 96 hours, ATP measurement was performed with Cell TITER-Glo Luminescent Cell Viability Assay (Promega) and the luminescence were measured with Perkin Elmer Victor 2 (GMI). Each test was conducted in triplicate.

### Sequencing of truncated RAFs

To identify RAF1 and BRAF truncation points in GTL-16 selected cell lines, we generated PCR products using pFB vector sense primer 5′-GGCTGCCGACCCCGGGGGTGG-3′ and antisense specific primers for C-terminal of target transcripts (murine and human RAF1 (NM_029780.3 and NM_002880.3 respectively) and murine Braf (NM_139294.5) respectively) and we cloned them into the pCR TOPO vector using the TOPO-TA Cloning Kit (Invitrogen). Sequencing was performed with TOPO TA M13 sense primer 5′-GTAAAACGACGGCCAG-3′ and antisense primer 5′-CAGGAAACAGCTATGAC-3′. All sequencing experiments were performed by automated sequencing by ABI Prism 3730 (Applied Biosystems).

### Western blotting

For Western blot the following antibodies were used: polyclonal antibody against human RAF1 (Upstate - Cat.# 07-396), polyclonal antibody against human and murine phosphorilated BRAF (Ser445) (Cell Signaling Technology - Cat.# 2696); monoclonal antibody against murine Raf1 (Santa Cruz Biotechnology - Cat.# sc-7267); and antibody against vinculin (Sigma - Cat.# V9131). Total cellular proteins were extracted by solubilizing the cells in boiling Laemmli buffer followed by sonication. Extracts were centrifuged at 12,000 rpm for 30 min and normalized with the BCA Protein Assay Reagent Kit (Pierce); 30 μg of lysates were run on 10% SDS-polyacrylamide gels, transferred onto nitrocellulose membranes (Hybond; GE Healthcare) and incubated with the antibodies overnight at 4°C. Nitrocellulose-bound antibodies were detected by the enhanced chemiluminescence (ECL) system (Promega).

### Xenograft experiments

All animal procedures were approved by the Ethical Committee of the Candiolo Cancer Institute and the Italian Ministry of Health. GTL-16 wild-type, GTL-16 human-delta-RAF1 and GTL-16 human-delta-BRAF cells (2 × 10^6^) were resuspended in 200ul of PBS and inoculated subcutaneously into the right posterior flanks of 6-week-old female CD-1 nu/nu mice (Charles River Laboratories). Mice were treated orally with 40 mg/kg/day of JNJ (the maximum tolerated dose) or vehicle. Treatment was initiated when the tumor volume reached approximately 50 mm^3^. Tumor volume was monitored every 3 days for 21 days.

## SUPPLEMENTARY MATERIAL FIGURES


