# Supplementary material for: Truncated RAF kinases drive resistance to MET inhibition in MET-addicted cancer cells
Source: Oncotarget. 2014 Nov 15;6(1):221–33. doi: 10.18632/oncotarget.2771 (PMC4381590; doi:10.18632/oncotarget.2771)
Supplement: Supplementary file 1 [file oncotarget-06-221-s001.pdf]

Truncated RAF kinases drive resistance to MET inhibition in MET-addicted cancer cells

Supplementary Material

Full-length retroviral cDNA library transduction.

Retroviral expression libraries were purchased as ViraPort Mouse Retroviral Library Supernatants (Stratagene, La Jolla, CA, USA). For determining cell transduction efficiency, target cells were transduced with a standard green fluorescent protein (GFP) retroviral supernatant (pFB-hrGFP) of comparable titer to that of the libraries. In particular $5x10^4$ cells were seeded onto 6-well tissue culture plates. The following day 1ml dilutions from 10^{-1} to 10^{-5} of retroviral supernatant in complete growth medium supplemented with 10µg/ml DEAE-dextran (Amersham Bioscence) were added to each well. After 3 hours, an additional ml of complete growth medium was added to each well. GFP expression analysis was performed after 48 hours by trypsinization, fixation in 1% paraformalhdeide-2% FBS and flow cytometry on a FACS Calibur flow cytometer (Becton Dickinson). The titer of the library, expressed as GFP transduction units (TU) per ml, was calculated as (10^5) x (fraction of GFP+) x (dilution). GTL-16 library transduction experiments were performed by plating $5x10^5$ cells in 100 mm plates. The following day, viral supernatants, diluted for a multiplicity of infection (MOI) ~ 2 in 3 ml of complete medium supplemented with 10µg/ml DEAE-dextran, were added. After 3 hours at 37°C, additional 7ml of growth medium were added to each plate. After 48 hours, plates were split in three, one for microarray analysis, one for freezing and one for expansion and selection.

RNA extraction and processing for xenoarray analysis.

RNA was extracted using the miRNeasy kit (Qiagen), according to the manufacturer's protocol. The quantification and quality analysis of RNA was performed on a Bioanalyzer 2100 (Agilent). Synthesis of cDNA and biotinylated cRNA was performed using the Illumina TotalPrep RNA Amplification Kit (Ambion), according to the manufacturer's protocol, with the following variations to optimize xenoarray analysis: library-specific cDNA synthesis: 20 µg of total RNA were used with 4 pmol of a pFB-specific primer (T7-pFB, sequence: GGCCAGTGAATTGTAATACGACTCACTATAGGGAGG CGGCGAACCCCAGAGTCCCGCTCA, HPLC-purified from Sigma) with standard reaction conditions. The T7-pFB primer contains the T7 promoter (for cRNA synthesis) followed by a vector specific sequence, which is present in the 3' region of all transcripts derived from the library. This procedure is able to retrotranscribe selectively only the library transcripts. Quality assessment and quantification of cRNAs were performed on Bioanalyzer 2100.

Hybridization of GTL-16 derived cRNAs on Illumina Mouse WG-6_V1.1 arrays (Cat. n. BD-26-112) was carried out for 18 hours according to the manufacturer's protocol, using 1.5 µg of T7-pFB-derived cRNA. These arrays contain about 48k probes exploring the transcripts contained in the Refseq database (with an additional 11,603 probes from Mouse Exonic Evidence Based Oligonucleotide (MEEBO) set), and therefore more reliable. Hybridization of GTL16-derived cRNAs infected with human libraries were carried out on Illumina Human-HT12_V4 arrays (Cat. n. BD-103-02-04), using 750 ng of T7-dT(24)- derived cRNA. These arrays contain about 48k probes.

Array washing was performed using Illumina High-stringency wash buffer for 30 min at 55°C (while 10 min for human arrays) followed by staining and scanning according to standard Illumina protocols. Probe intensity and detection data were obtained using the Illumina BeadStudio software (Genome Studio).

Quantitative realtime PCR.

For RT-PCR, cDNA was synthesized using RT High Capacity cDNA kit (Applied Biosystems), and PCR primers were designed with Primer Express software (Applied Biosystems) against mouse and human specific regions of the transcripts (except for the *PGK1* housekeeping gene): human *PGK1* (NM_000291.3) sense 5'-AGCTGCTGGGTCTGTCATCCT-3' and antisense 5'-TGGCTCGGCTTTAACCTTGT-3'; murine *Pgk1* (NM_008828.2): sense 5'-CAGATTGTTTGGAATGGTCCTG-3' and antisense 5'-CCCCTAGAAGTGGCTTTCACC-3';

human *RAF1* (NM_002880.3) N-terminal sense 5'-TGGAAGACGATCAGCAATGG-3' and antisense 5'-GGCGCTGATAGCCAAACTG-3'; human *RAF1* C-terminal sense 5'-AGTAAAGTCACGCTGGAGTGGTT-3' and antisense 5'-TGGGTTGTTATCCTGCATTCG-3'; mouse *Raf1* (NM_029780.3) N-terminal sense 5'-TGCGTCGGATGCGAGAAT-3' and antisense 5'-GAGGAAGGGCTGGAGGTGTT-3'; mouse *Raf1* C-terminal sense 5'-ACAGACAGCTCAGGGAATGGA-3' and antisense 5'-TTTCACCGTGAGGCCTTCAT-3'; human *BRAF* (NM_004333.4) C-terminal sense 5'-TGTTACCCAGTGGTGTGAGG-3' and antisense 5'-GACCCACTCCATCGAGATTT-3'; mouse *Braf* (NM_139294.5) N-terminal sense 5'-CATTCCTGAAGAGGTATGGAATATCA-3' and antisense 5'-

GGGTTATGCTCTCCACCAAATT-3’; mouse *Braf* C-terminal sense 5’-ACAGCACCCACACCTCAACA-3’ and antisense 5’-TAGAATAGCCCATGAAAAGGAGGAT-3’.

Cycle condition performed: 2' at 50° C, 10’ at 95° C, followed by 40 cycles (15'' at 95° C and 1' at 60° C) performed on 7900 HT (Applied Biosystems).

454 Sequencing data generation.

RNA Extraction and retroviral vector-specific RNA synthesis.

RNA was extracted from GTL-16 HsS1-UNS, HsS2-UNS, HsS1-SEL and HsS2-SEL cells using miRNeasy Mini kit (Qiagen) according to the manufacturer’s protocol. Quantification and quality analysis of RNA was performed, respectively, with the Nanodrop 1000 Spectrophotometer and the Bioanalyzer 2100 with RNA 6000 Nano kit (Agilent Technologies). Synthesis of pFB-specific first strand cDNA was performed using 20 ug of total RNA, 8 pmol of T7-pFB primer (sequence: 5’ GGCCAGTGAATTGTAATACGACTCACTATAGGGAGGCGGCGAACCCCAGAGTC CCGCTCA 3’, HPLC-purified, Sigma-Aldrich), 10U AMV reverse transcriptase and its 1x Buffer (Fermentas) at 50°C for 2h. Second strand cDNA and cRNA synthesis was performed using Illumina TotalPrep RNA amplification kit (Ambion) according to the manufacturer’s protocol. Quantification and quality analysis of cRNA was performed with the same methods as the total RNA described above.

cDNA libraries synthesis for 454 sequencing.

Double strand cDNA synthesis and purification was performed starting from 200 ng of cRNA according to GS FLX Titanium cDNA Rapid Library Preparation method (Roche) but skipping the RNA fragmentation step. After the second strand synthesis,

we introduced a “fill-in” reaction in order to maximize the polymerization and obtain fully double-stranded DNA filaments. This step was carried out with the following mix and cycle: 1X dNTPs, 3.6 mM $MgCl_2$, 1X Buffer, 2.5U Fast Start Taq (Roche) at 95°C 10’, (95°C 30’’, 72°C 2’) 10 cycles, 72°C 10’. Fill-in products were purified using 1.6X Ampure XP beads (Beckman Coulter). End repair, adaptors ligation, small fragment removal and library quantification were performed according to the above protocol. To differentiate each sample GS FLX Titanium Rapid Library MID Adaptors (numbers 1-4, Roche) were used.

emPCR and sequencing procedures.

To set emPCR conditions for 454 sequencing we performed an emPCR titration with 12.5, 25, 50 copies per beads (cpb) using the GS-FLX Titanium SV emPCR kit (Lib L, Roche). Then we chose the optimal cpb for each sample in order to reach 8% enriched beads as suggested by Roche protocol. Libraries from selected and unselected samples were mixed with 1:4 ratio before the final emPCR performed with GS-FLX Titanium LV emPCR kit (Lib L, Roche). Sequencing run was performed using GS-FLX Titanium sequencing kit XLR 70 (Roche) and GS-FLX PicoTiterPlate kit 70X75 (Roche) on a GS FLX 454 sequencer (Roche). We divided the PicoTiterPlate into two regions both containing two samples with a different MID Adaptor.

All the alignments were performed with Blast on pFB from and *RAF1* (NM_002880.3) sequences.

ProteinQuest analysis

ProteinQuest (BioDigitalValley) performs an accurate search, in PubMed database, as it lets to refine the field of interest by selecting specific dictionaries/ontologies. To find truncated forms of *RAF1* protein reported in SDS-Page and Western Blot images we interrogated this platform with the query: “("RAF1"[All Fields] OR "V-RAF-1 MURINE LEUKEMIA VIRAL ONCOGENE HOMOLOG 1"[All Fields] OR "CRAF"[All Fields] OR "NS5"[All Fields] OR "RAF-1"[All Fields]) AND "loattrfree full text"[sb]” (found 1615 publications). Subsequently all images obtained (1421) were extracted and analyzed by specific logic filters: (i) presence of *RAF1* text in the images (OCR recognition); (ii) selection images with more immunoreactive bands OR with reported molecular weight; (iii) images with more than one molecular weight; (iv) finally, images with more immunoreactive bands AND more than one molecular weight (25 images).

The image analysis has been performed according to the following steps: (i) Extraction of the "Y" coordinates in pixel of the molecular weight and the signal apex of the immunoreactive band; (ii) Processing the logarithmic curve of the molecular weight distribution in relation to the pixels image; (iii) After building the curve for each image the value of molecular weight of the immunoreactive bands were obtained by measuring the "Y" coordinates in pixels of the signal apex of each band; (iv) All the immunoreactive bands were ordered by molecular weight in a matrix that highlights the relative abundance of each band respect to the total number of analyzed samples; (v) Finally, a virtual gel was created for each image and all gels were added together in a single virtual gel image (Supplementary Fig. S3).

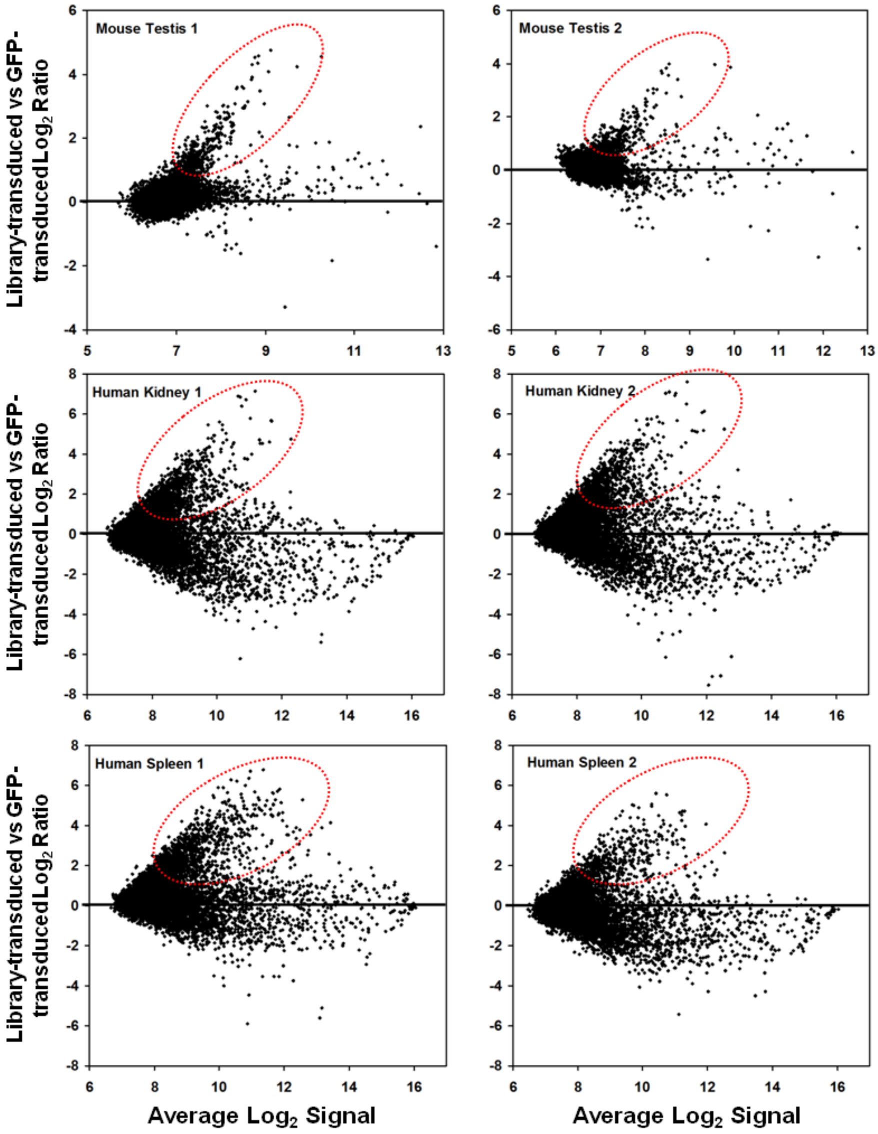

Supplementary Figure 1: Detection by xenoarray analysis of expression library-derived transcripts in transduced cells. The MA plots display, for each probe of the array, on the y-axis the differential signal (Log2 ratio) between cells transduced with the various libraries (as indicated) and with GFP; on the x-axis the average signal of GFP- and library- transduced cells. The dashed red line indicates library-derived transcripts. The lower background observed in mouse testis library-transduced cells is due to the use of murine arrays, which greatly reduces the signal of host cell-derived human transcripts.

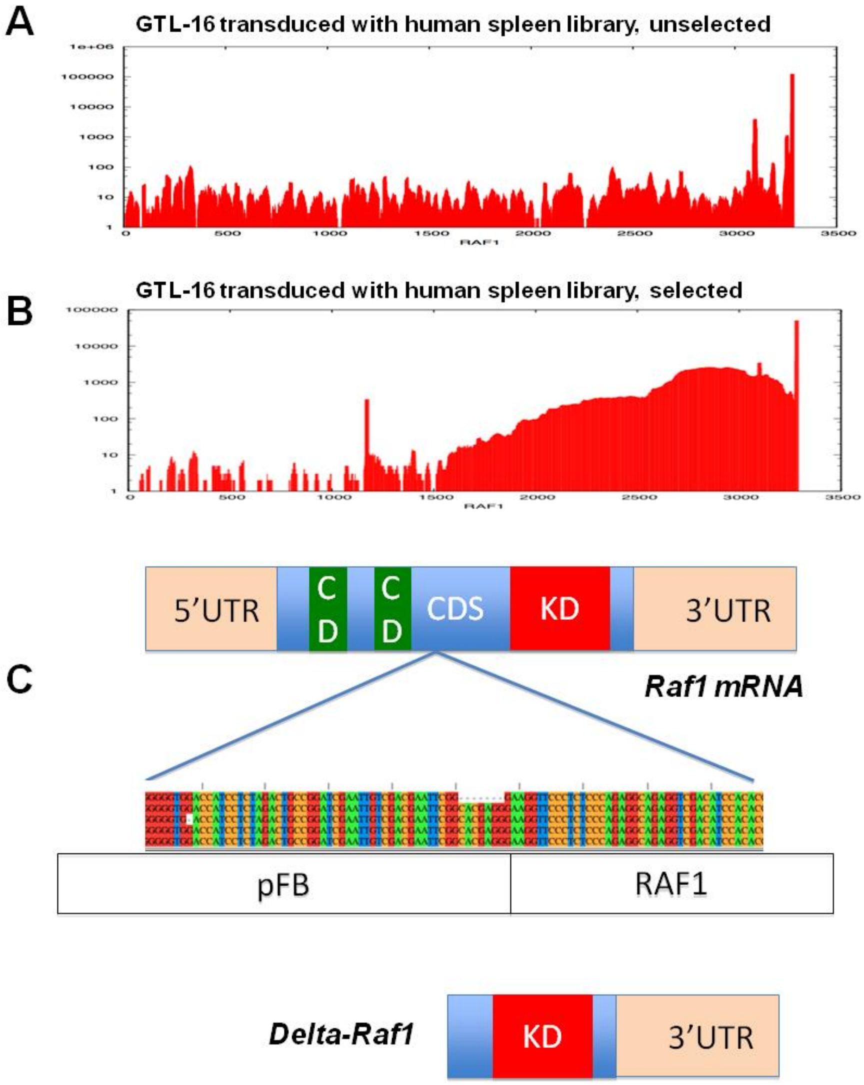

Supplementary Figure 2: Deep sequencing confirms enrichment upon PHA selection of retroviral library-derived truncated *RAF1* transcripts in GTL-16 cells. (A) Coverage of the *RAF1* transcript in the unselected cell population. (B) Coverage of the *RAF1* transcript in the PHA-selected cell population. The 3'-half of the transcript is much more represented. (C) In the selected population, four reads were mapped both on *RAF1* and vector (pFB) sequence; all the reads correspond to the very same *RAF1*-pFB construct. Graphical representation of truncated form of *RAF1* after selection: the transcript maintained the Kinase Domain, but lost both regulatory domains.

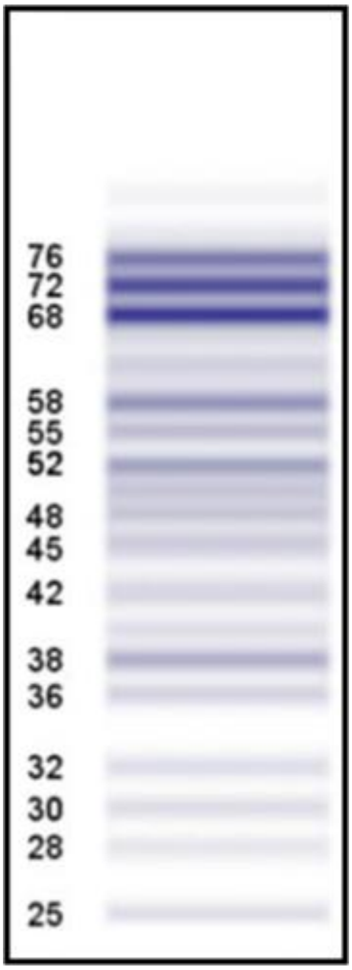

Supplementary Figure 3: Systematic detection of truncated RAF1 proteins in published data using ProteinQuest. Virtual Western Blot image of RAF1 in which the band density is proportional to the frequency of observation of the corresponding variant.

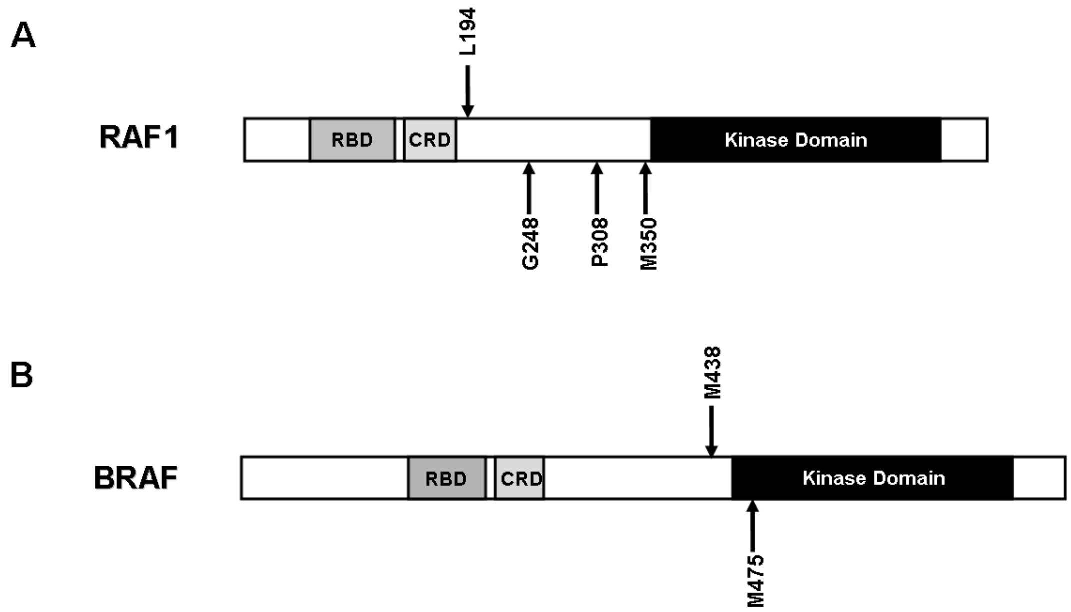

Supplementary Figure 4: Details of truncated constructs. (A) Schematic diagram of protein domain structure of human RAF1 (648 aa) showing the truncation positions of library derived delta proteins enriched in GTL-16 cells during MET inhibitor resistance aquisition (M350, P308 and G248 in MmT, HsK and HsS respectively) and in tumors described by Palanisamy et al. (L194). (B) Schematic diagram of protein domain structure of human BRAF (766 aa) showing the truncation position of MmT library derived delta *Braf* enriched in murine *Raf1*-shRNA3-GTL-16 during MET inhibitor resistance acquisition (M475) and in tumors described by Palanisamy et al. (M438).

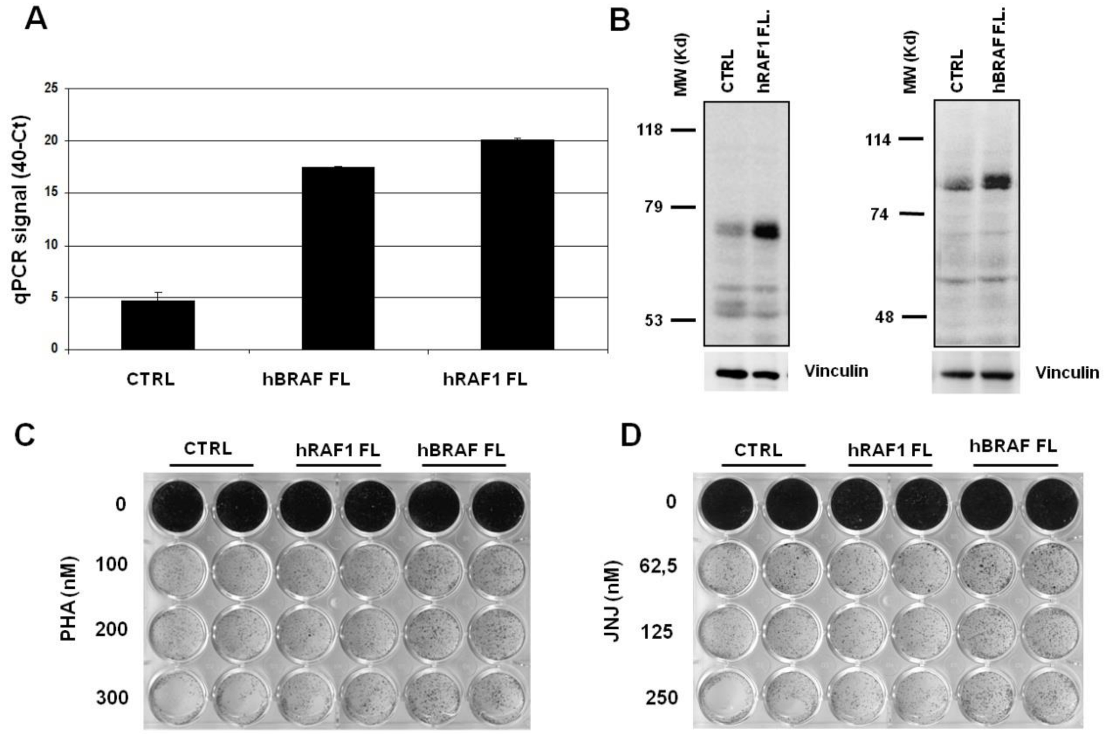

Supplementary Figure 5: Expression of full-length RAF1 or BRAF is ineffective. (A) qPCR validation on GTL-16 cells transduced with human FL-RAFs. (B) Western Blot analysis on GTL-16 WT cells (CTRL) and transduced with human full length RAF1 or BRAF; vinculin was used as loading control. (C, D) GLT-16 cells transduced with human full length RAF1 or BRAF and untransduced cells (CTRL) were treated with increasing concentrations of (C) PHA or (D) JNJ (as indicated) for two weeks and subsequently fixed, stained, and photographed.

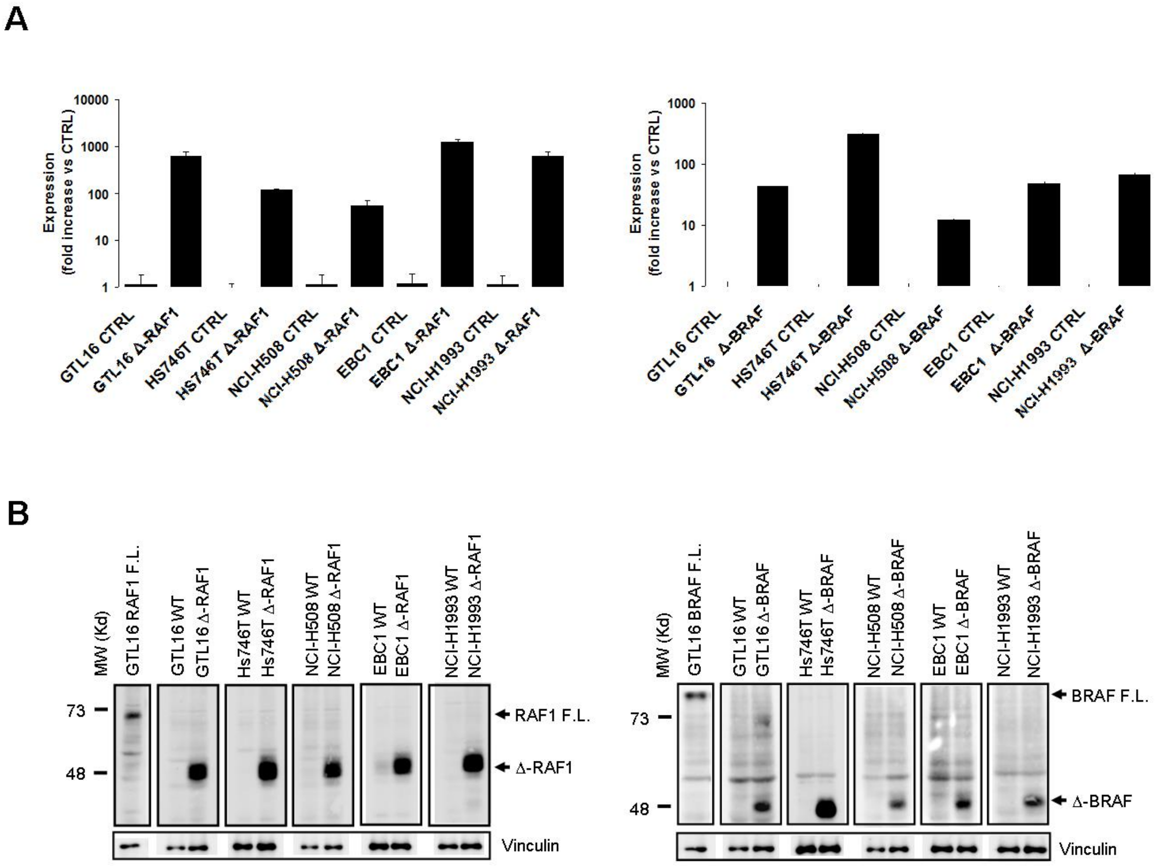

Supplementary Figure 6: Truncated RAFs expression in different addicted cancer cell lines.

(A) qPCR validation and (B) Western Blot analysis on GTL-16, Hs746T, NCI-H508, EBC1 and NCI-H1993 cells transduced with human truncated RAFs. The proteins were detected by an antibody against C-terminus of RAFs. GTL-16 cells transduced with human RAFs full-length were used as control for the full size protein detection and vinculin as loading control. The molecular weights of the Delta-RAF proteins correspond to the expected.

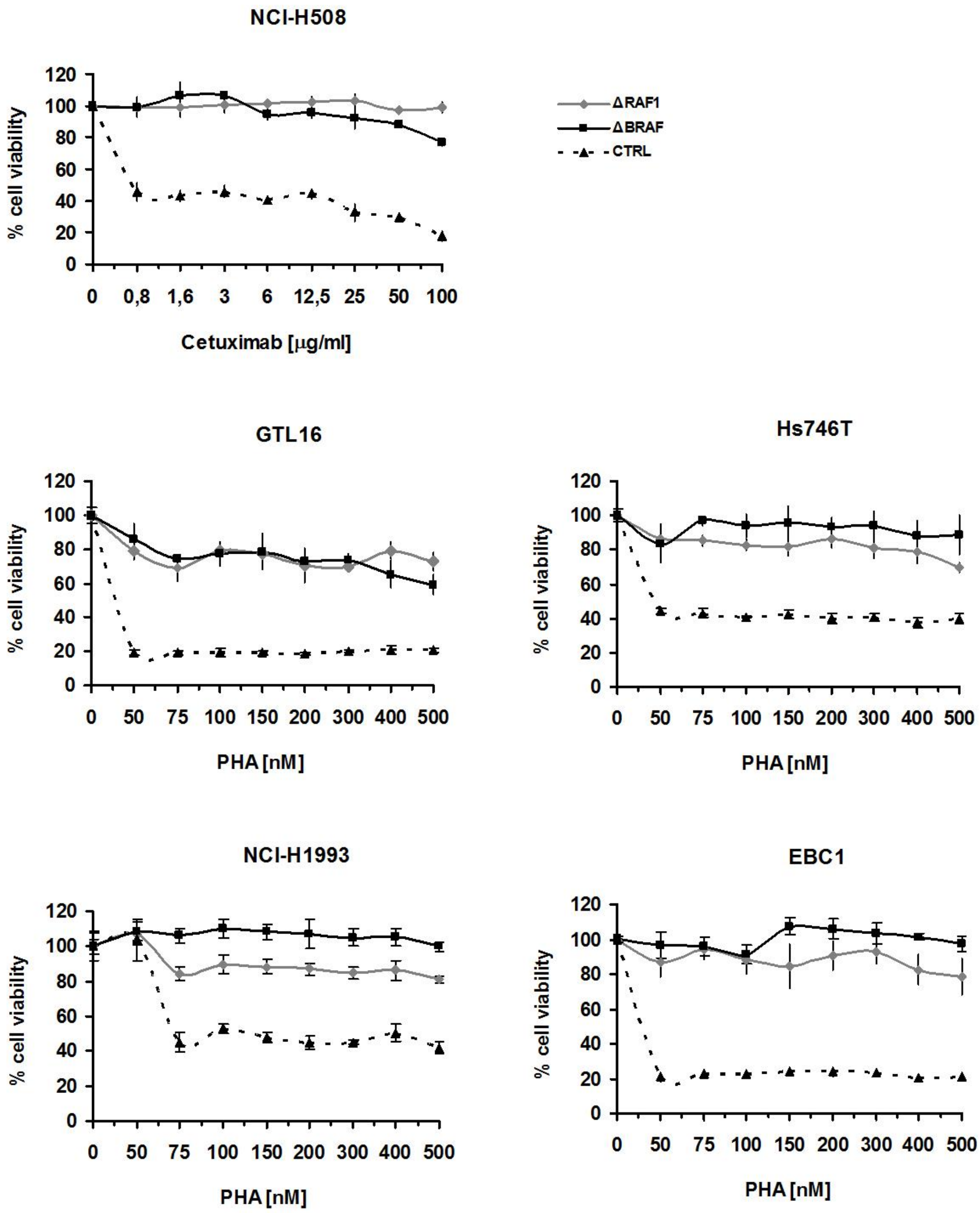

Supplementary Figure 7: Truncated RAFs confer resistance in different addicted cancer cell lines.

Cell-viability assay at increasing concentrations of PHA or Cetuximab on GTL16, Hs746T, EBC1, NCI-H1993 and NCI-H508 cells transduced with human truncated RAFs.

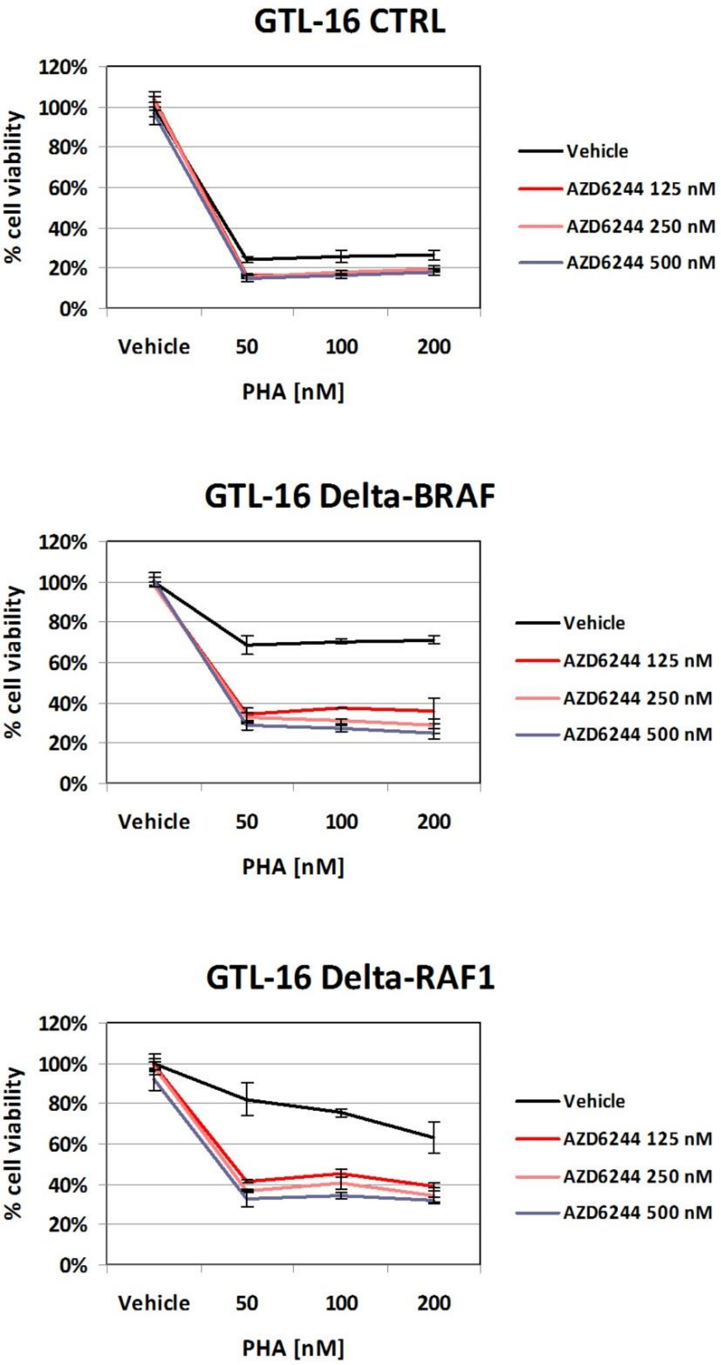

Supplementary Figure 8: MET-MEK inhibitors combination restore drug sensitivity in GTL-16 cells.

Cell-viability assay at different dosage combinations of PHA and AZD6244 on GTL-16 cells transduced with human truncated RAFs.
